# A retrospective cohort study evaluating healthcare resource utilization in patients with asthma in Japan

**DOI:** 10.1038/s41533-019-0128-8

**Published:** 2019-04-29

**Authors:** Hiromasa Inoue, Masanari Kozawa, Ki Lee Milligan, Minako Funakubo, Ataru Igarashi, Emil Loefroth

**Affiliations:** 1 Department of Pulmonary Medicine, Graduate School of Medical and Dental Sciences, Kagoshima University, Kagoshima, Japan; 2 Novartis Pharma K.K., Tokyo, Japan; 3 Department of Drug Policy and Management, The University of Tokyo, Tokyo, Japan; 4 Novartis Sweden AB, Taby, Sweden

**Keywords:** Outcomes research, Health care economics

## Abstract

Although the global economic burden of asthma is well described, detailed data regarding Asia, particularly for Japan, are relatively scarce. This retrospective study aims to fill this evidence gap by evaluating asthma-associated healthcare resource utilization (HCRU) and economic burden in Japanese patients aged ≥16 years, identified using anonymized patient data from the Japan Medical Data Center (JMDC) database from April 2009 to March 2015. Asthma severity was classified according to asthma treatment guidelines from the Japanese Society of Allergology. HCRU was calculated based on hospitalizations, emergency room visits, outpatient visits, and prescriptions. Incidence rate ratios (IRRs) for HCRU and per-patient-per-year direct costs were reported. In addition, differences across HCRU and cost variables for severe versus non-severe asthma patients were also compared. Of 541,434 asthma cases identified from the JMDC database during the study period, 54,433 patients who met the inclusion criteria were included in this analysis. HCRU and costs were heavily concentrated within severe asthma, a subgroup comprising 12.7% of total study population. Moreover, patients with severe asthma had significantly higher all-cause hospitalizations, outpatient visits, outpatient prescriptions (IRR [95% CI], 1.60 [1.46–1.76]; 1.43 [1.41–1.45]; 1.24 [1.22–1.25], respectively), and total medical costs (mean ± SD costs, US$ 4345 ± 11,104 versus US$ 1528 ± 3989, *P* < 0.001 (*t*-test); US$ 1 = 110 JPY) compared with those with non-severe asthma. The burden of asthma is significantly and disproportionately concentrated in Japanese severe asthma patients, suggesting clinical failure to achieve adequate disease control. This study highlights the unmet needs for severe asthma in Japan and provides a catalyst for important dialogues in advancing public health.

## Introduction

Asthma is a major chronic, non-infectious respiratory disease affecting more than 350 million people worldwide.^1^ In Asia, the prevalence of asthma is 3–7% and is expected to rise.^2,3^ In Japan, ~3 million people suffer from asthma, making it the leading chronic respiratory disease; severe asthma accounts for 7% of Japanese patients with asthma, and moderate asthma for 30%.^4,5^ The burden of asthma in Asia is in line with global trends.^6,7^ A cross-sectional observational study conducted in six countries from the Asia-Pacific region demonstrated considerable healthcare resource utilization (HCRU) and associated cost burden owing to chronic respiratory diseases (combined: asthma, allergic rhinitis, chronic obstructive pulmonary disease (COPD), and rhinosinusitis).^8^ The socioeconomic burden for asthma, specifically in Asia, consists of individual reports from Thailand, South Korea, Singapore, Taiwan, and India.^9–14^ However, data focusing on HCRU in asthma patients from Japan are scarce. The Japan Medical Data Center (JMDC) Claims Database contains claims from insurance societies throughout Japan, whose population consists of employees and their family members (includes children and elderly relative beneficiaries who are not employed).^15^ The study of treatment patterns and HCRU insurance claims may enhance awareness of asthma-related treatment decisions in real-world practice, and inform Japan’s investments in future health policy. Therefore, the present study aimed to evaluate healthcare utilization and economic burden of asthma in Japan as well as to assess the impact of asthma severity (as defined by the Japanese Society of Allergology (JSA)^16^) on healthcare utilization.

## Results

### Patient disposition

**Fig. 1:**
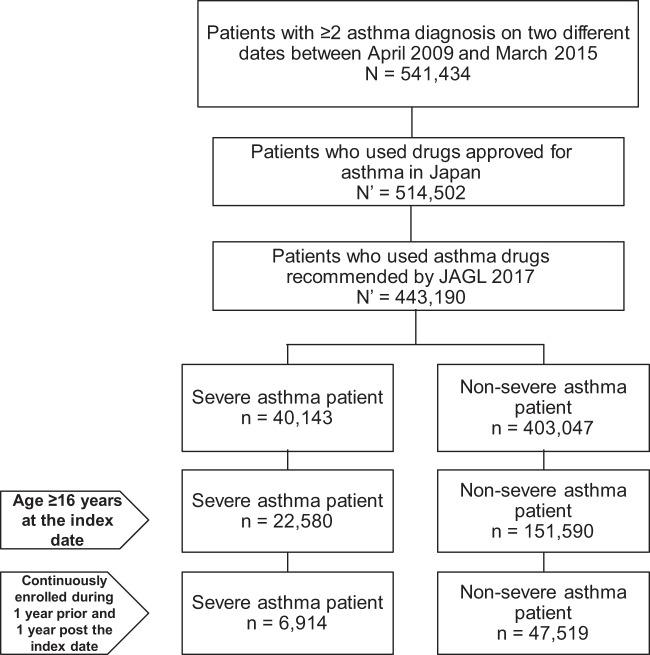
Patient disposition. JSA Japanese Society of Allergology

Of the 541,434 patients identified with asthma diagnosis from the JMDC database, 514,502 patients had confirmed prescriptions for asthma drugs approved in Japan (Supplementary Table 1). Of these, 40,143 patients received drugs recommended for JSA Step 4 severity, 403,047 patients received drugs recommended for Steps 1–3, and 71,312 patients were excluded as their prescription profiles for asthma did not meet the criteria for any treatment step. A total of 54,433 patients who met the inclusion criteria were included in the study; of these, 6914 (12.7%) were classified as severe asthma patients (JSA Step 4; Fig. 1).

### Baseline demographics and clinical characteristics

Patient demographics and baseline characteristics are presented in Table 1. The mean ± standard deviation (SD) age of patients in the severe asthma cohort (43.03 ± 12.20 years) was significantly higher (*P* < 0.0001 (*t*-test)), compared with the non-severe asthma cohort (39.76 ± 11.98 years). There was a slightly higher proportion of female patients, compared with men in both cohorts; however, no significant differences in gender distribution were observed by asthma severity. Occurrence of comorbidities was significantly higher in severe asthma cohort versus non-severe asthma cohort, with recurrent respiratory infection being the most frequent comorbidity in both cohorts.

**Table 1:** Patient demographics and baseline characteristics

Characteristic	Severe asthma cohort (*N* = 6914)	Non-severe asthma cohort (*N* = 47,519)	*P* value
Age, years	43.03 ± 12.20	39.76 ± 11.98	<0.0001
Gender, *n* (%)			
Men	3150 (45.6)	22,115 (46.5)	0.127
Women	3764 (54.4)	25,404 (53.5)	
Member type, *n* (%)			
Employee	4069 (58.9)	29,212 (61.5)	<0.0001
Family	2845 (41.1)	18,307 (38.5)	
Quan–Charlson comorbidity index	1.82 ± 2.02	1.08 ± 1.45	<0.001
Comorbidities, *n* (%)			
Recurrent respiratory infection	4329 (62.6)	25,627 (53.9)	<0.001
Rhinitis/allergic rhinitis	4046 (58.5)	19,774 (41.6)	<0.001
Eczema	2357 (34.1)	12,344 (26.0)	<0.001
Sinus disease	2015 (29.1)	8571 (18.0)	<0.001
Gastroesophageal reflux	936 (13.5)	3707 (7.8)	<0.001
Pneumonia	922 (13.3)	3095 (6.5)	<0.001
Psychiatric disorder (depression, anxiety)	710 (10.3)	3259 (6.9)	<0.001
Cardiovascular disease (myocardial infarction, stroke, heart failure)	587 (8.5)	2178 (4.6)	<0.001
Osteoporosis	244 (3.5)	665 (1.4)	<0.001
Diabetes	238 (3.4)	942 (2.0)	<0.001
Inflammatory bowel disease	169 (2.4)	506 (1.1)	<0.001
Chronic obstructive pulmonary disease	109 (1.6)	195 (0.4)	<0.001
Obstructive sleep apnea	100 (1.4)	507 (1.1)	0.013
Hyperglycemia	65 (0.9)	241 (0.5)	<0.001
Nasal polyps	48 (0.7)	71 (0.1)	<0.001
Food allergy and anaphylaxis	40 (0.6)	161 (0.3)	0.004

Data presented as mean ± SD unless otherwise specified

*SD* standard deviation

*P* values were calculated using *χ*^2^ test for categorical variables, *t*-test for continuous variables

### Healthcare resource utilization

Overall, HCRU due to asthma and all-cause was high at baseline and the 12-month follow-up in both the cohorts. Patients in the severe asthma cohort had significantly higher mean HCRU in terms of hospitalizations, emergency room (ER) visits, outpatient visits, and prescriptions, compared with the non-severe asthma cohort at baseline and at the 12-month follow-up (all *P* < 0.001 (*t*-test); Table 2).

**Fig. 2:**
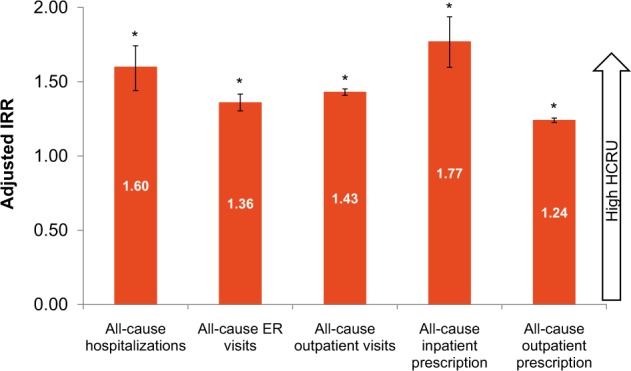
Incidence rate ratios of HCRU events for severe versus non-severe asthma patients. Data presented as IRR (95% CI). Error bars represent 95% CI values. *P < 0.001 (*P* values were calculated using *χ*^2^ test for categorical variables, *t*-test for continuous variables). IRR is the ratio of incidence rates of all-cause HCRU events in severe and non-severe asthma patients. IRR adjusted for age, gender, insurance type, institution type, baseline comorbidity of interests, and Charlson index score. CI confidence interval, ER emergency room, HCRU healthcare resource utilization, IRR incidence rate ratio

Incidence rate ratios (IRRs) of HCRU events were significantly higher in the severe cohort, compared with the non-severe cohort (*P* < 0.001); these included higher incidences of all-cause hospitalizations, ER visits, inpatient and outpatient prescriptions, and outpatient visits (Fig. 2).

**Table 2:** Healthcare resource utilization (all-cause and asthma related) at baseline and follow-up

	Baseline (12 months before index date)			Follow-up (12 months after index date)		
	Severe asthma cohort (*N* = 6914)	Non-severe asthma cohort (*N* = 47,519)	*P* value	Severe asthma cohort (*N* = 6914)	Non-severe asthma cohort (*N* = 47,519)	*P* value
Number of hospitalizations						
All cause	0.2 ± 0.5	0.1 ± 0.3	<0.001	0.2 ± 0.8	0.1 ± 0.3	<0.001
Number of patients, *n* (%)	820 (11.9)	2535 (5.3)		791 (11.4)	2447 (5.1)	
Asthma related	0.0 ± 0.2	0.0 ± 0.1	<0.001	0.1 ± 0.3	0.0 ± 0.1	<0.001
Number of patients, *n* (%)	135 (2.0)	146 (0.3)		301 (4.4)	394 (0.8)	
Length of stay, days						
All cause	3.1 ± 18.1	0.6 ± 5.8	<0.001	2.7 ± 18.3	0.7 ± 11.1	<0.001
Number of patients, *n* (%)	820 (11.9)	2535 (5.3)		791 (11.4)	2447 (5.1)	
Asthma related	0.4 ± 5.5	0.0 ± 2.0	<0.001	1.0 ± 8.9	0.1 ± 3.7	<0.001
Number of patients, *n* (%)	135 (2.0)	146 (0.3)		301 (4.4)	394 (0.8)	
Number of ER visits						
All cause	1.3 ± 3.6	0.8 ± 2.3	<0.001	1.8 ± 5.2	1.1 ± 2.8	<0.001
Number of patients, *n* (%)	2868 (41.5)	15,721 (33.1)		3304 (47.8)	20,212 (42.5)	
Asthma related	0.3 ± 1.4	0.1 ± 0.8	<0.001	0.6 ± 2.1	0.4 ± 1.3	<0.001
Number of patients, *n* (%)	981 (14.2)	2,699 (5.7)		1605 (23.3)	9420 (19.8)	
Number of outpatient visits						
All cause	19.0 ± 22.1	10.9 ± 13.9	<0.001	27.0 ± 26.2	14.5 ± 15.6	<0.001
Number of patients, *n* (%)	6753 (97.7)	44,823 (94.3)		6910 (99.9)	47,515 (100.0)	
Asthma related	5.0 ± 9.4	1.4 ± 4.2	<0.001	9.6 ± 11.7	4.7 ± 6.6	<0.001
Number of patients, *n* (%)	4266 (61.7)	15,295 (32.2)		5964 (86.3)	44,914 (94.5)	
Number of inpatient prescription						
All cause	0.2 ± 0.7	0.1 ± 0.4	<0.001	0.3 ± 1.1	0.1 ± 0.4	<0.001
Number of patients, *n* (%)	692 (10.0)	2508 (5.3)		983 (14.2)	2589 (5.4)	
Asthma related	0.0 ± 0.3	0.0 ± 0.1	<0.001	0.1 ± 0.5	0.0 ± 0.2	<0.001
Number of patients, *n* (%)	135 (2.0)	137 (0.3)		298 (4.3)	387 (0.8)	
Number of outpatient prescription						
All cause	9.3 ± 6.1	6.1 ± 5.0	<0.001	9.1 ± 3.5	6.4 ± 3.6	<0.001
Number of patients, *n* (%)	6707 (97.0)	43,813 (92.2)		6909 (99.9)	47,515 (100.0)	
Asthma related	3.3 ± 4.5	1.0 ± 2.3	<0.001	6.2 ± 5.5	3.4 ± 3.3	<0.001
Number of patients, *n* (%)	4242 (61.4)	15,140 (31.9)		5951 (86.1)	44,882 (94.5)	

The index date was defined as the first date of the highest asthma stage being recorded for a patient

Data presented as mean ± SD, unless otherwise stated, *ER* emergency room, *SD* standard deviation

*P* values were calculated using *χ*^2^ test for categorical variables and *t*-test for continuous variables

### Economic burden—unadjusted and adjusted medical costs

**Fig. 3:**
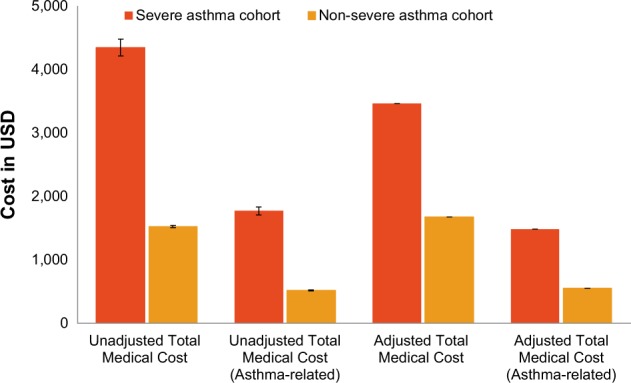
Mean adjusted and unadjusted total medical costs for severe versus non-severe asthma patients. Data presented as mean ± standard error. Error bars represent standard errors. 1 USD = 110 JPY

The high HCRU was also reflected from the medical expenditure on these resources. Mean total direct medical costs were significantly higher in the severe asthma cohort than in the non-severe asthma cohort at baseline and the 12-month follow-up (*P* < 0.001; (*t*-test) Table 3). Furthermore, the medical costs were high in patients in severe asthma cohort relative to those in the non-severe asthma cohort after adjusting for demographics (Fig. 3).

**Table 3:** Mean unadjusted medical cost (all-cause and asthma related) at baseline and follow-up

Cost in USD	Baseline (12 months before index date)			Follow-up (12 months after index date)		
	Severe asthma cohort (*N* = 6914)	Non-severe asthma cohort (*N* = 47,519)	*P* value	Severe asthma cohort (*N* = 6914)	Non-severe asthma cohort (*N* = 47,519)	*P* value
Total cost of hospitalization						
All cause	1564.8 ± 11,286.08	232.5 ± 2144.06	<0.001	1277.9 ± 8775.80	318.2 ± 3082.74	<0.001
Asthma related	177.8 ± 2551.09	13.2 ± 486.52	<0.001	397.2 ± 3562.31	52.8 ± 1388.20	<0.001
Total cost of ER visits						
All cause	94.8 ± 240.84	56.6 ± 159.70	<0.001	139.2 ± 341.68	79.2 ± 201.72	<0.001
Asthma related	27.2 ± 119.10	7.9 ± 62.65	<0.001	53.4 ± 162.30	27.5 ± 119.63	<0.001
Total cost of outpatient visits						
All cause	1334.8 ± 3401.99	645.7 ± 1507.01	<0.001	2102.3 ± 4704.60	867.8 ± 1738.74	<0.001
Asthma related	425.0 ± 2210.63	88.5 ± 555.85	<0.001	789.0 ± 2605.50	298.1 ± 1024.23	<0.001
Total cost of inpatient prescription						
All cause	211.6 ± 2652.77	16.8 ± 374.36	<0.001	435.3 ± 1697.08	27.6 ± 563.00	<0.001
Asthma related	40.9 ± 936.26	1.1 ± 66.30	<0.001	88.8 ± 2179.86	238.3 ± 624.43	<0.001
Total cost of outpatient prescription						
All cause	898.9 ± 2711.46	321.1 ± 986.43	<0.001	1712.5 ± 4068.71	502.3 ± 1167.14	<0.001
Asthma related	385.4 ± 1629.08	63.3 ± 379.24	<0.001	868.2 ± 2179.86	238.3 ± 624.43	<0.001
Total medical cost						
All cause	3404.6 ± 12,506.55	1083.6 ± 3055.00	<0.001	4345.3 ± 11,104.16	1528.0 ± 3989.21	<0.001
Asthma related	838.5 ± 3586.13	145.1 ± 913.08	<0.001	1772.5 ± 4960.14	519.6 ± 1889.91	<0.001

The index date was defined as the first date of the highest asthma stage being recorded for a patient

1 USD = 110 JPY; data presented as mean ± SD

*ER* emergency room, *SD* standard deviation

*P* values were calculated using *χ*^2^ test for categorical variables and *t*-test for continuous variables

## Discussion

The present study offers real-world evidence on the significant socio-economic burden of asthma in Japan, and confirms a disproportionate concentration of HCRU and asthma-related cost burden in Japanese patients with severe asthma. We identified the main driver of asthma HCRU in Japan to be Step 4 treatment (applied as surrogate marker for severe asthma). Additionally, advanced age and comorbidities were enriched in the severe asthma cohort. This observation suggests that severe asthma patients not only suffer from chronic asthma but also may have a compromised state of health related to multiple factors.

Severe asthma patients constituted 12.7% of the overall asthma population, and were significantly older compared with non-severe asthma patients (43.03 versus 39.76 years; *P* *<* 0.0001), with 4.4% and 2.2% of patients in the 65- to 74-year-old age group, respectively. Patients within the higher age ranges may represent senior employees or elderly members covered by the employee’s family health insurance policy; the proportions of working and non-working seniors is unknown, making a deeper interpretation challenging. Of note, gender differences did not influence any of the study outcomes.

Comorbidities, including infections, allergic rhinitis, sinus disease, and gastroesophageal reflux, which are all known to be associated with uncontrolled asthma were presented more frequently in severe versus non-severe asthma cohort, with pneumonia presenting twice as frequently. On the other hand, COPD was noted in a few patients from both cohorts and the small numbers limited useful interpretation; these patients were not excluded from the analysis to investigate all comorbidities related to asthma. Furthermore, with advancing age, overlapping features of asthma and COPD may become increasingly present.^17^ However, the study was not designed to evaluate the relationship between asthma severity and comorbidities.

HCRU was significantly higher in severe asthma patients compared with non-severe patients. Interestingly, increase in IRR and medical costs in the follow-up year were found in both severe and non-severe asthma patients, implying a high medical attendance burden shared across all Japanese patients with asthma. Mean adjusted and unadjusted medial costs were significantly higher in severe asthma patients than in non-severe asthma patients. The healthcare settings from most to least utilized were outpatient visits (the great majority), ER attendance, and finally hospitalization, an expected pattern considering Japan’s universal health access. Direct costs provide a partial picture of the disease burden. While this study did not capture indirect costs, one could argue that indirect costs contribute significantly, especially in a working-age population taking into account work productivity losses, quality-of-life deficits, caregiver burdens, and also in special cases of severe asthma, unintended side effects of glucocorticoid therapy.

In recent times, the use of secondary databases such as JMDC has demonstrated utility in assessing drug utilization in various disease areas.^18–20^ To the best of our knowledge, this is the first study to describe HCRU in adult asthma patients, conducted using a large Japanese population insurance claims database. Results from our study are consistent with prior international evidence. A study by Sullivan et al retrospectively evaluated economic and healthcare utilization as well as sociodemographic characteristics of severe asthma patients in the US from a claims database.^21^ Findings from that study were consistent with those from our study; the proportion of severe asthma patients was low, but this subgroup contributed majorly to the economic burden. A report by McDonald et al also highlighted that the care required for the relatively small proportion of severe asthma patients has a major impact on the overall healthcare system.^22^ A Japanese prospective study in more than 3600 severe allergic asthma patients, on omalizumab therapy, showed different medical care patterns compared with our study, with approximately 20% and 26% of patients receiving asthma-related care in the hospital and ER, respectively, which was higher than that observed in our study. However, the population demographics of that study differed with respect to age and gender: elevated mean age (59.3 years) with 44% of patients aged >65 years, and more female patients (64.5%).^23^ A comprehensive look at asthma control (which was out of scope for this analysis) considers reducing symptoms, airflow limitation, and exacerbation risks by optimizing inhaler techniques, trigger avoidance, adherence, perception of disease, and appropriateness of therapy versus need for escalation.^24^ In the current study, due to its retrospective design using the secondary database, data on above clinical outcomes (e.g., symptoms, lung function) could not be collected, and therefore step-up in therapy offers a reasonable, though incomplete, surrogate measure for loss of disease control in patients in the non-severe asthma cohort.

Strengths of this study include HCRU data generation in a large Japanese population, characterization of severe and non-severe asthma cohorts, and confirmation of age and comorbidities as relevant variables for asthma outcomes. The disposition for 70,000 of ~540,000 total eligible patients was unclear, because these 70,000 patients received prescriptions that were not as per guideline recommendations. This preliminary observation deserves further attention to examine if these patients had only mild intermittent asthma or if they were persistent asthma patients whose treatment regimens could be further optimized. The results highlight a potential deviation from consensus care guidelines, and present an opportunity for further examining real-world clinical practices, especially in an era of biologics and precision medicine when both evidence based and individualized therapies offer novel solutions.

Limitations include retrospective design with its inherent limits, insurance claims are not ideal for clinical questions, variable accuracy for capturing comorbidities, lack of visibility for patient-initiated treatments, off-label use, or over-the-counter alternatives. Drug treatment as a proxy of disease severity is commonly used in commercial claims database analyses and is an acceptable method of classifying patients into disease severity categories.^16,25^ However, asthma severity also considers clinical outcomes that could not be measured in our study, including airflow limitation, intensity of asthma symptoms, or experience of exacerbations. Japanese life expectancy is higher than the world average and a limited representation of patients aged >65 years could reduce the generalizability of findings to the overall population. Smoking behavior and body mass index have a major impact on asthma severity; however, these variables and others that are important for understanding asthma treatment response, and asthma control, were not included in our analysis.

Overall, the study offers novel real-life evidence in adult patients with asthma in Japan and highlights the need for initiatives to optimize patient care. Appropriate treatment policies, through existing and innovative therapeutic strategies must be designed and implemented to improve clinical outcomes and to reduce HCRU in asthma patients.

The burden of asthma is significantly and disproportionately concentrated in Japanese severe asthma patients, suggesting clinical failure to achieve adequate disease control. This study enhances awareness of current unmet needs in Japan for asthma and provide novel evidence to engage public and private sector stakeholders toward future health solutions.

## Methods

### Study design and patients

**Fig. 4:**
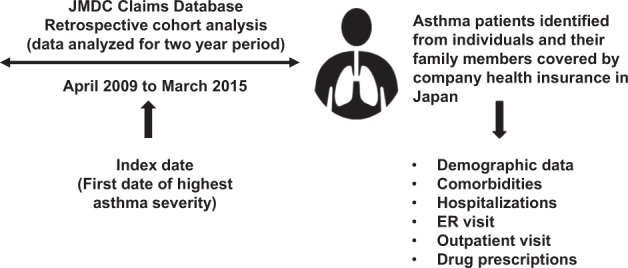
Study design

This was a retrospective, cohort study in asthma patients from Japan, performed using the JMDC database for the study period from April 2009 to March 2015 (Fig. 4). An asthma case was defined as any patient claim in the database identified with the disease code J45 (asthma) or J46 (status asthmaticus) as per ICD-10 at minimum two different months during the study period. Asthma severity was defined according to the JSA guidelines^16^ recommended drug therapies, with Step 4 corresponding to severe asthma, and Steps 1–3 corresponding to non-severe asthma. Two cohorts were established for comparison, the severe asthma cohort (Step 4 patients) and the non-severe asthma cohort (Steps 1–3 patients). Categorization of asthma patients in Steps 1–4 based on JSA recommended therapies is described in Supplementary Table 2.

Patients aged ≥16 years with a diagnosis of asthma during the study period were included in the analysis. Eligible patients included employees and their family members covered by their company health insurance plan. They were required to have been enrolled continuously in the database for a minimum of 1 year before (baseline) and 1 year after the index date. The index date was defined as the first date of the highest asthma stage, as per JSA guideline recommendations, recorded for a patient during the study period. Data were collected for a period of 2 years.

The study was approved by the Clinical Research Review Network of the Japan Review Board, Osaka, Japan. This study was conducted in accordance with the Guidelines for Good Pharmacoepidemiology^26^ Practices of the International Society for Pharmacoepidemiology and with the ethical principles laid down in the Declaration of Helsinki. The study analysis was approved by JMDC. Patient identity was not disclosed. The study is reported in accordance with Strengthening the Reporting of Observational Studies in Epidemiology (STROBE) recommendations^27^ (Supplementary Table 3).

### Data source

The JMDC database is the largest public dataset for patient health in Japan and is compiled by health insurance records covering 1.45 million employees and their family members from medium- to large-scale companies.^15^ The JMDC database consists of pharmacy and medical claims, and includes demographic data such as year of birth, sex, year and month of medical service or prescription provided, diagnosis based on ICD-10 codes, drug names and dosages, inpatient or outpatient, size of hospital, type of claim, and annual company health check-up data. Retirees aged >65 years generally drop out of the insurance system at that age; however, elderly patient claims exist, and it is unknown whether they represent senior employees or parents of employees covered under the family insurance plan.

### Study outcomes

Asthma-specific HCRU was characterized and estimated at baseline and at 12 months after the index date in patients classified in severe and non-severe asthma cohorts based on disease severity. All-cause-related HCRU was also compared in these groups of patients at baseline and at 12 months post index date. Asthma-related and all-cause HCRU was characterized by number of hospitalizations, days in hospitals, prescriptions, ER visits, and outpatient office visits during the study period. IRRs of all-cause HCRU events were adjusted for age, gender, insurance type, institution type, baseline comorbidity of interests, and Charlson index score, and were compared between cohorts. HCRU-associated total costs, which included ER costs, inpatient and outpatient costs, and outpatients’ prescription drug costs, were also estimated in these patients. Demographic variables (age, gender, family type), clinical characteristics, and type of medical institution were measured at index date or the closest time before index date.

### Statistical analysis

Mean and SD were presented for continuous variables; number of cases and percentages were presented for categorical variables. For categorical variables, *χ*^2^ tests were performed to determine significant differences; for continuous variables, the Wilcoxon rank sum test and the unequal variance two-sample *t*-test were performed. All clinical characterization variables were measured at the time of (or at the time closest to but not exceeding) the index date. The HCRU profiles were characterized by assessments at baseline and at the 12-month follow-up, and comparisons were made between all-cause and asthma-related utilization. IRRs were evaluated using a negative binomial model for count of HCRU events, and presented with 95% CI. Cost was analyzed using a two-part model that included gamma regression following a logistic model and was presented as mean (±SD). Total medical costs were adjusted for age, gender, insurance type, institution type, baseline count outcome, baseline cost, and Charlson index score by using multiple regression models. Statistical analyses were performed using SAS studio 3.4.

### Reporting Summary

Further information on research design is available in the Nature Research Reporting Summary linked to this article.

## Supplementary information

Supplementary Material.

Life Sci Reporting Summary

## Data Availability

Novartis is committed to sharing access to patient-level data and supporting documents from eligible studies with qualified external researchers. These requests are reviewed and approved by an independent review panel on the basis of scientific merit. All data provided are anonymized to respect the privacy of patients who have participated in the trial in line with applicable laws and regulations.

## Supplementary information

**Supplementary information** accompanies the paper on the *npj Primary Care Respiratory Medicine* website (10.1038/s41533-019-0128-8).
