# Supplementary material for: A retrospective cohort study evaluating healthcare resource utilization in patients with asthma in Japan
Source: NPJ Prim Care Respir Med. 2019 Apr 29;29:13. doi: 10.1038/s41533-019-0128-8 (PMC6488614; doi:10.1038/s41533-019-0128-8)
Supplement: Supplementary file 1 — Supplementary Material. [file 41533_2019_128_MOESM1_ESM.docx]

**Title page**

**A retrospective cohort study evaluating healthcare resource utilization in patients with asthma in Japan**

**Running title: Healthcare utilization in asthma patients in Japan**

Hiromasa Inoue ^1^, Masanari Kozawa^2^, Ki Lee Milligan^2^, Minako Funakubo^2^, Ataru Igarashi^3^, Emil Loefroth^4^

^1^Department of Pulmonary Medicine, Graduate School of Medical and Dental Sciences, Kagoshima University, Kagoshima, Japan; ^2^Novartis Pharma K.K., Tokyo, Japan; ^3^Department of Drug Policy and Management, The University of Tokyo, Tokyo, Japan; ^4^Novartis Sweden AB, Taby, Sweden

**Corresponding author:**

Prof. Hiromasa Inoue

Department of Pulmonary Medicine

Graduate School of Medical and Dental Sciences

Kagoshima University

Kagoshima, Japan

Phone: +81-099-275-6476

Email: inoue-pulm@umin.net

**Disclosure:** The study was funded by Novartis Pharma K.K. (Toranomon Hills, Mori Tower 1-23-1, Toranomon, Minato-ku, Tokyo 105-6333, Japan)

**Supplementary Table 1 List of asthma drugs recommended by JAGL 2017**

| Drug category | Drug name |
| --- | --- |
| Inhaled corticosteroid | Beclomethasone dipropionate |
|  | Fluticasone propionate |
|  | Budesonide |
|  | Ciclesonide |
|  | Mometasone furoate |
| Inhaled corticosteroid/ β_2_-adrenergic agonist | Salmeterol xinafoate/Fluticasone propionate |
|  | Budesonide/formoterol |
|  | Fluticasone propionate/formoterol |
|  | Fluticasone furoate/vilanterol trifenatate |
| Anti-IgE antibody | Omalizumab |
| Oral corticosteroid | Hydrocortisone |
|  | Hydrocortisone sodium succinate |
|  | Prednisolone |
|  | Prednisolone sodium succinate |
|  | Methylprednisolone |
|  | Methylprednisolone acetate |
|  | Methylprednisolone sodium succinate |
|  | Triamcinolone |
|  | Triamcinolone acetate |
|  | Dexamethasone |
|  | Betamethasone |
| Xanthine derivative | Theophylline |
|  | Aminophylline |
|  | Diprophylline |
|  | Proxyphylline |
|  | Diprophylline |
|  | Proxyphylline |
| Bronchodilator | Salmeterol Xinafoate |
|  | Salbutamol sulfate |
|  | Terbutaline sulfate |
|  | Tulobuterol hydrochloride |
|  | Procaterol hydrochloride |
|  | Fenoterol hydrobromide |
|  | Clenbuterol hydrochloride |
|  | Adrenaline |
|  | Isoprenaline sulfate |
|  | Isoprenaline hydrochloride |
|  | Trimetoquinol hydrochloride |
|  | Ipratropium bromide |
|  | Oxitropium bromide |
|  | Tiotropium bromide hydrate |
| Leukotriene receptor antagonist | Pranlukast hydrate |
|  | Zafirlukast |
|  | Montelukast sodium |
| Anti-allergy | Sodium cromoglicate |
|  | Tranilast |
|  | Amlexanox |
|  | Ibudilast |
|  | Pemirolast potassium |
| Histamine blocker | Ketotifen fumarate |
|  | Azelastine hydrochloride |
|  | Oxatomide |
|  | Mequitazine |
|  | Epinastine hydrochloride |
| Thromboxane α2 synthesis inhibitor | Ozagrel hydrochloride hydrate |
| Thromboxane α2 synthesis inhibitor | Seratrodast |
| Th2 inhibitor | Suplatast tosilate |
| IgE, immunoglobulin E; JAGL, Japanese Guideline for the Diagnosis and Treatment of Allergic Diseases | |

**Supplementary Table 2 Categorization of asthma patients in JSA Steps 1–4 based on recommended therapies**

| Step 4 asthma patients | - Patients with: - High dose ICS and minimum of two controllers from LABA, LTRA, Theo or TIO. The use of high dose ICS and the controllers should have a ≥1-day overlap - Continuous use of oral corticosteroid for more than 29 days (with 14 days grace period) - Omalizumab |
| --- | --- |
| Step 2–3 asthma patients | - Patients with: - Low-/medium-/high-dose ICS and maximum of one controller from LABA, LTRA, Theo or TIO - Low-/medium-dose ICS use and minimum of two controllers - The use of ICS and the controllers should have a ≥1-day overlap - Patients identified as Step 4 and Step 1 were excluded |
| Step 1 asthma patients | - Patients with: - One of the following: low-dose ICS, LTRA or Theo - Patients identified as Step 4 were excluded |
| ICS, inhaled corticosteroid; JSA, Japanese Society of Allergology; LABA. Long-acting β_2_-agonist; LTRA, leukotriene receptor antagonist; Theo, theophylline; TIO, tiotropium | |

**Supplementary Table 3 STROBE Statement—checklist of items that should be included in reports of observational studies**

|  | | Item No | Recommendation |
| --- | --- | --- | --- |
| Title and abstract | | 1 | (*a*) Indicated the study’s design with a commonly used term in the title or the abstract |
|  |  |  | (*b*) An informative and balanced summary of what was done and what was found is provided in the abstract |
| Introduction | | | |
| Background/rationale | | 2 | Explained the scientific background and rationale for the investigation being reported |
| Objectives | | 3 | Stated specific objectives, no prespecified hypotheses defined for this study |
| Methods | | | |
| Study design | | 4 | Key elements of study design presented early in the paper |
| Setting | | 5 | Described the setting, database and data collection |
| Participants | | 6 | Included eligibility criteria as well as the sources and methods of selection of participants |
| Variables | | 7 | Clearly defined all outcomes |
| Data sources/ measurement | | 8 | For each variable of interest, provided sources of data and details of methods of assessment (measurement) |
| Bias | | 9 | Data collected retrospectively from the JMDC database; no potential selection bias identified |
| Study size | | 10 | Explained study size calculation |
| Categorical variables | | 11 | Explain how variables were handled in the analyses; quantitative variables not applicable |
| Statistical methods | | 12 | Described all statistical methods |
| Results | | | |
| Participants | 13 | Reported the numbers of individuals at each stage of study — numbers required for study, included in the study and analyzed | |
| Descriptive data | 14 | Included characteristics of study participants (e.g., demographic, clinical, social) | |
|  |  | Indicated number of participants with missing data for each variable of interest | |
| Outcome data | 15 | Reported numbers of outcome events or summary measures | |
| Main results | 16 | Provided estimates and statistical significance | |
| Other analyses | 17 | Report other analyses performed — e.g., analyses of subgroups and interactions, and sensitivity analyses—not applicable | |
| Discussion | | | |
| Key results | 18 | Summarized key results with reference to study objectives | |
| Limitations | 19 | Discussed limitations of the study. Discuss both direction and magnitude of any potential bias | |
| Interpretation | 20 | Included a cautious overall interpretation of results considering objectives, limitations | |
| Generalizability | 21 | Discussed the generalizability (external validity) of the study results | |
| Other information | | | |
| Funding | 22 | Provided the source of funding for the present study | |
